# Long-term clinical outcomes of catheter ablation in patients with atrial fibrillation predisposing to tachycardia-bradycardia syndrome: a long pause predicts implantation of a permanent pacemaker

**DOI:** 10.1186/s12872-018-0834-0

**Published:** 2018-05-30

**Authors:** Dong-Hyeok Kim, Jong-Il Choi, Kwang No Lee, Jinhee Ahn, Seung Young Roh, Dae In Lee, Jaemin Shim, Jin Seok Kim, Hong Euy Lim, Sang Weon Park, Young-Hoon Kim

**Affiliations:** Division of Cardiology, Department of Internal Medicine, Korea University College of Medicine and Korea University Medical Center, 73, Inchon-ro, Seongbuk-gu, Seoul 02841 Republic of Korea

**Keywords:** Atrial fibrillation, Tachycardia, Bradycardia, Catheter ablation, Pacemaker

## Abstract

**Background:**

There is a controversy as to whether catheter ablation should be the first-line therapy for tachycardia-bradycardia syndrome (TBS) in patients with atrial fibrillation (AF).

**Methods:**

We aimed to investigate long-term clinical outcomes of catheter ablation in patients with TBS and AF. Among 145 consecutive patients who underwent catheter ablation of AF with TBS, 121 patients were studied.

**Results:**

Among 121 patients, 11 (9.1%) received implantation of a permanent pacemaker during a mean 21 months after ablation. Length of pause on termination of AF was significantly greater in patients who received pacemaker implantation after ablation than those who underwent ablation only (7.9 ± 3.5 vs. 5.1 ± 2.1 s, *p* < 0.001). Using a multivariate model, a long pause of 6.3 s or longer after termination of AF was associated with the requirement to implant a permanent pacemaker after ablation (HR 1.332, 95% CI 1.115-1.591, *p* = 0.002).

**Conclusion:**

This study suggests that, in patients with AF predisposing to TBS, long pause on termination of AF predicts the need to implant a permanent pacemaker after catheter ablation.

**Electronic supplementary material:**

The online version of this article (10.1186/s12872-018-0834-0) contains supplementary material, which is available to authorized users.

## Background

Tachycardia-bradycardia syndrome (TBS) is literally a two-fold disease that is characterized by prolonged sinus pause on termination of atrial tachyarrhythmias, including atrial fibrillation (AF). Implantation of a permanent pacemaker plus antiarrhythmic drug (AAD) prescription is the mainstay therapy for patients with TBS due to sinus pause or its aggravation on AAD [1]. However, AF-related problems (e.g. AF symptoms, progression to persistent AF [2], tachycardia-mediated cardiomyopathy [3, 4], AAD use, anticoagulation) may remain even after implantation of a permanent pacemaker. Furthermore, device-related complications (e.g. infection, endocarditis, vascular complications, need for generator change) may also occur.

Catheter ablation has been widely performed in patients with AF, and its clinical benefits and safety in patients with AF have been well documented. Catheter ablation is also known to be curative for TBS, especially in PV-triggered AF [5], through elimination of triggers for tachycardia. Recent studies demonstrated that ablation, compared to pacemaker implantation, decreased tachycardia-related hospitalization and was effective at controlling AF and prolonged sinus pause [6]. However, long term follow-up data are needed because some populations of patients are likely to have intrinsic sinus node dysfunction (SND) even in the clinical setting of TBS, and SND can gradually progress in those patients who require a pacemaker after catheter ablation of AF [7, 8]. Thus, whether catheter ablation should be considered the first line therapy for TBS in AF remains debated. In this study, we investigated the long-term clinical outcomes of catheter ablation in patients with TBS on termination of AF. Furthermore, we determined predictors for triage of patients in whom catheter ablation is expected to be more beneficial than implantation of a permanent pacemaker.

## Methods

### Patient population

**Fig. 1:**
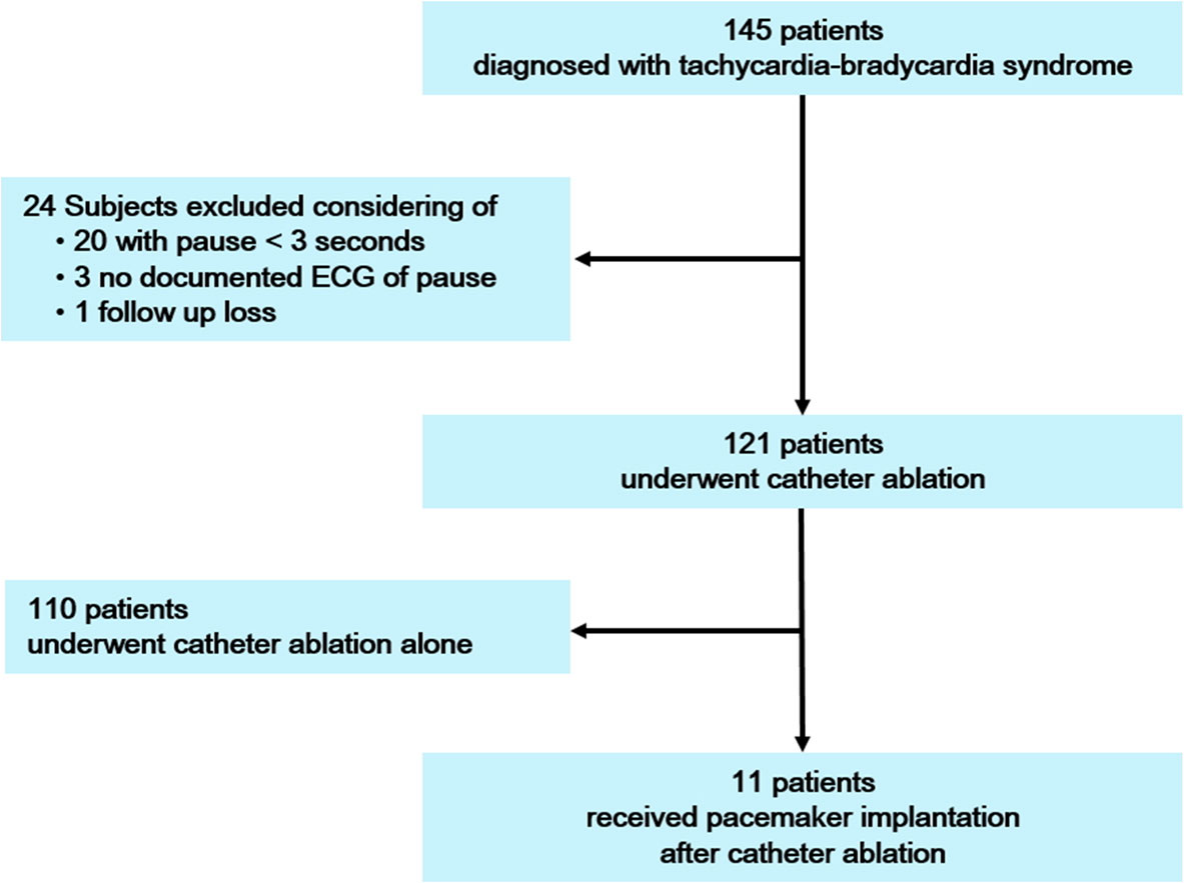
Study population and flow chart

Figure 1 shows the study populations. Patients who visited Korea University Medical Center and underwent catheter ablation of AF or pacemaker implantation during June 2004-June 2015 were retrospectively examined. Definitions of AF type and catheter ablation of AF followed the Guidelines for the management of atrial fibrillation and the 2014 consensus documents of the American Heart Association/American College of Cardiology/Heart Rhythm Society [9, 10]. TBS was defined as in previous studies, namely a ventricular pause following termination of atrial tachyarrhythmia (e.g. AF) [11, 12]. TBS was defined when more than 3 s of sinus pause was documented on ECG immediately after termination of AF leading to related symptoms, such as dizziness and syncope. If long sinus pause more than 3 s after termination of tachyarrhythmia could not be documented, we checked Holter ECG or event ECG recorder repeatedly. Although ECG with long pause more than 3 s was documented, TBS was not diagnosed if there were no symptoms related to the ECG documentation. Catheter ablation of AF in patients with AF and TBS was determined at the physicians’ discretion based on symptoms of palpitations, dizziness, syncope, and history of stroke.

Among the 145 patients, 24 patients were excluded; 20 with a pause of less than three seconds, 3 because of no documented electrocardiography (ECG) of pause, and 1 due to follow-up loss. Finally, 121 patients with catheter ablation were studied. A permanent pacemaker was implanted in 11 patients who were highly symptomatic due to a long pause after ablation. This study was approved by the institutional review board in Korea University Medical Center.

### Procedures for catheter ablation

After written informed consent was obtained, all the patients underwent electrophysiology study and catheter ablation. Prior to the procedure, all antiarrhythmic drugs were discontinued, and more than 5 half-lives were allowed to pass before the study was performed. Amiodarone was discontinued at least 1 month before the ablation procedure. All catheters were inserted via the femoral vein. A duodecapolar catheter (St. Jude Medical Inc., Lowell, MA, USA) was placed in the coronary sinus (CS) to record both the low right atrium (RA) and CS electrograms, and a decapolar catheter (Bard Electrophysiology Inc., Lowell, MA, USA) was positioned at the high RA. A quadripolar catheter was placed at either the His bundle or superior vena cava (SVC). Intracardiac electrograms were recorded using a Prucka CardioLab™ electrophysiology system (General Electric Health Care System Inc., Milwaukee, WI, USA) or EP Workmate system (EP MedSystem, Inc./St. Jude Medical Inc., St. Paul, MN, USA). After double transseptal puncture, the patients were administered anticoagulants such as intravenous heparin to maintain an activated clotting time of between 300 and 400 s. Three-dimensional geometries of the LA and PVs were reconstructed using Ensite-NavX mapping system (St. Jude Medical Inc., St. Paul, MN, USA). Trigger was defined as initiation of AF before PV isolation. When non-PV trigger was detected, and then was also ablated [13, 14]. Circumferential pulmonary vein isolation (CPVI) with electrical PV isolation was performed. When AF followed CPVI, either linear ablation or complex fractionated atrial electrogram-guided ablation was performed additionally. When AF converted into atrial tachycardia (AT), AT was ablated according to the mechanisms of AT. For focal AT, RF energy was delivered at the focus; for macroreentrant AT, a line of block was created at the critical isthmus. The endpoints of the ablation were AF or AT termination. Each radiofrequency energy application was performed using an open-irrigated ablation catheter with a maximum temperature of 48 °C and a power of 25-35 W.

### Outcome measurements and patient follow-up

Primary outcome measurement was freedom from atrial tachyarrhythmia(s), AF or AT, after the procedures. After ablation, patients were asked to visit the outpatient clinic at 1, 3, 6, 9, and 12 months and then every 6 months thereafter or whenever they experienced tachycardia-related symptoms. ECG was performed at every visit. Holter monitor recording was performed in patients who were thought to have arrhythmia-related intermittent symptoms. Recurrence of atrial tachyarrhythmia was defined as an event lasting more than 30 s after a 3-month blanking period. Antiarrhythmic drugs (AADs) were taken during the first 3 months after the ablation. Discontinuation of AADs was determined at the physicians’ discretion.

### Statistical analysis

All values are expressed as means ± SD or as numbers and percentages where appropriate. Categorical data were compared by the χ^2^ test. Continuous variable data were compared by independent samples t-test when the distribution was normal or by the Mann-Whitney test if it the distribution was not normal. Kaplan-Meier analysis with the log-rank test was used to determine the probability of freedom from recurrent atrial tachyarrhythmia. Receiver operating characteristic (ROC) analysis was used to calculate sensitivity and specificity, and the area-under-the-curve (AUC) was used to compare accuracy for different lengths of pause. Cox regression analysis was used for the predictor model. Variables were selected on the basis of univariate significance. *P* < 0.05 was considered statistically significant. Statistical analyses were performed using SPSS Statistics 19.0 software (SPSS Inc., Armonk, NY, USA).

## Results

### Clinical characteristics

Total 121 patients underwent catheter ablation and clinical characteristics at baseline are summarized in Table 1. Mean longest pause following termination of AF were 5.4 s. After ablation, anticoagulant and antiplatelet therapy was continued according to CHADS_2_ score or CHA_2_DS_2_-VASc score: warfarin 37.2%, anti-platelet drug 49.6%, and NOAC 3.3% (Table 1).

**Table 1:** Demographics and clinical characteristics of the study participants

Factors	*n* = 121
Age, years old	61.1 ± 10.4
Male, n (%)	64 (52.9)
Longest pause, seconds	5.4 ± 2.4
Time of AF symptom onset, months	36.5 ± 32.3
Type of persistent AF, n (%)	17 (14.0)
AAD before procedure, n (%)	77 (63.6)
Class I drug	66 (54.5)
Class III drug	11 (9.1)
Antithrombotic drug, n (%)	109 (90.1)
Warfarin	45 (37.2)
Anti-platelet drug	60 (49.6)
NOAC	4 (3.3)
LVEF	57.8 ± 7.9
LA size, mm	41.0 ± 5.6
E/e′	9.6 ± 5.2
Hypertension, n (%)	66 (54.5)
Diabetes mellitus, n (%)	16 (13.2)
CHAS_2_DS_2_-VASc score	1.9 ± 1.3
0, n (%)	15 (12.4)
1, n (%)	39 (32.2)
≥ 2, n (%)	67 (55.4)
HAS-BLED score	1.6 ± 1.1

Values are expressed as means±SDs and numbers (percentages). *AF* atrial fibrillation, *AAD* anti-arrhythmic drug, *NOAC* non-vitamin K antagonist anticoagulant, *LVEF* left ventricular ejection fraction, *LA* left atrium

### Complications after ablation and clinical outcomes

After AF ablation, 9 complications were noted: cardiac tamponade (*n* = 6, 4.9%), groin hematoma (*n* = 2, 1.6%), and atrial esophageal fistula (*n* = 1, 0.8%). One patient (0.8%) died among total 121 patients. Four patients (3.3%) experienced stroke.

### Recurrence of atrial tachyarrhythmia after catheter ablation

After catheter ablation, the rate of any atrial tachyarrhythmia (AF or AT) recurrence is 19.0% (23 of 121) during mean 29.3 months of follow up. We investigate factors affecting recurrence according to age and trigger sites. Thirty-one patients (25.6%) were 70-year-old or more. Atrial tachyarrhythmia recurrence was not significantly different between patients with 70-year-old or more and those with younger than 70-year-old (18.9% vs. 19.4%, log-rank test *p* = 0.732). During the ablation procedures, triggers were identified in 73 patients (60.3%) and no trigger was identified in 48 patients (39.7%). There was no significant difference of atrial tachyarrhythmia recurrence between patients with triggers and those with no trigger (17.8% vs. 20.8%, log-rank test *p* = 0.559, Additional file 1: Figure S1). Among total patients, sixty-three patients (52.1%) had a pulmonary vein (PV) trigger; left superior PVs, left inferior PVs, right superior PVs, right middle PV, right inferior PV, and multiple PVs accounted for 27 (22.3%), 7 (5.8%), 13 (10.7%), 1 (0.8%), 2 (1.7%), and 13 (10.7%) trigger sites, respectively. Ten patients (8.3%) had non-PV triggers; eight (6.6%) at SVC and two (1.7%) at the high RA septum. There was no significant difference of atrial tachyarrhythmia recurrence between patients with PV trigger and those with non-PV trigger (18.6% vs. 14.3%, log-rank test *p* = 0.817, Additional file 2: Figure S2).

### Pacemaker implantation after catheter ablation

**Fig. 2:**
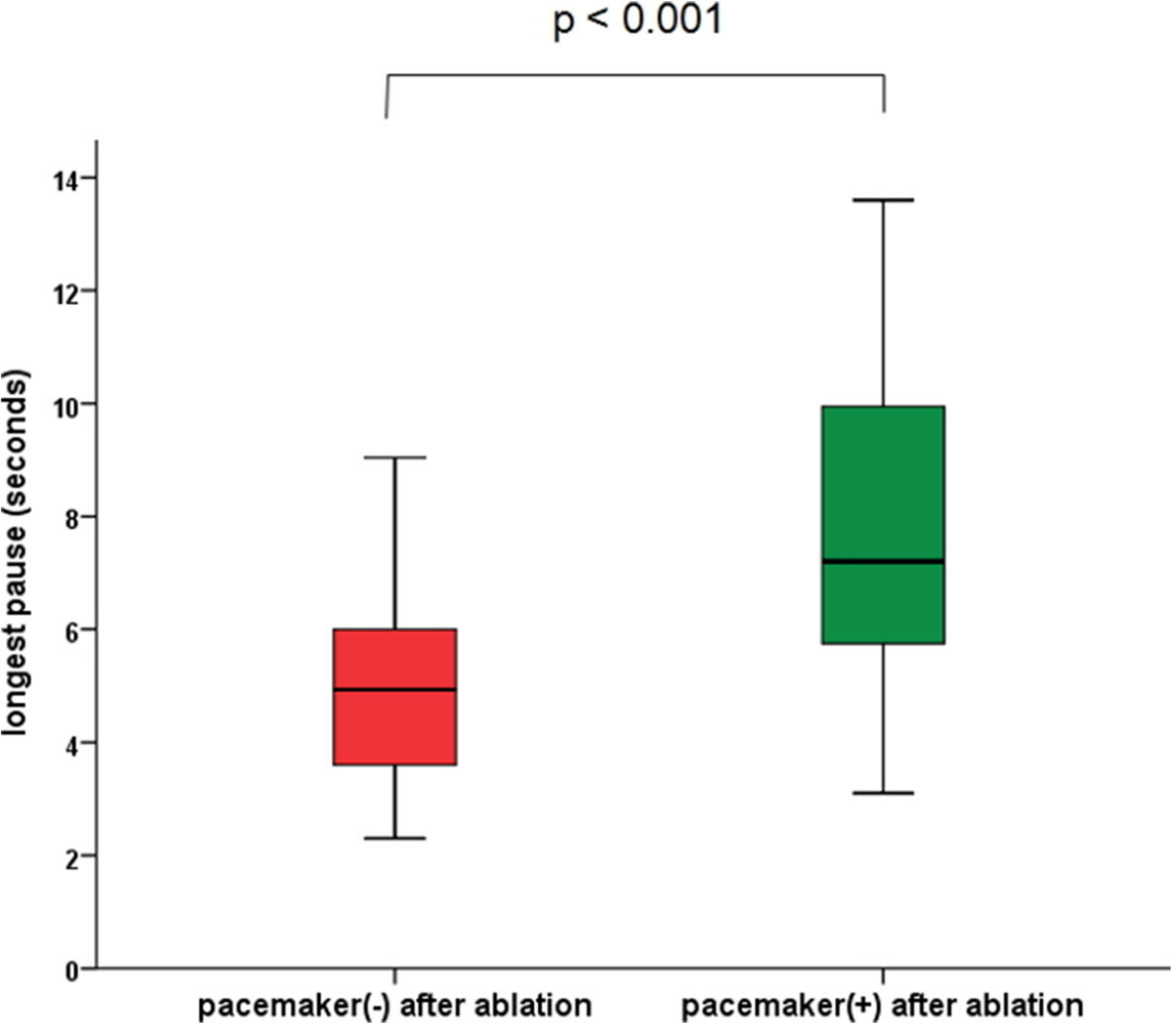
Comparison and the longest pause between patients who did not receive a pacemaker and those who received a pacemaker after catheter ablation

Following catheter ablation of AF, eleven patients (9.1%) received implantation of permanent pacemaker. Mean time interval from catheter ablation to pacemaker implantation was 21 months. The patients’ characteristics are shown in Table 2. Mean longest pause on termination of AF prior to catheter ablation was significantly longer in patients who underwent pacemaker implantation after catheter ablation compared to those who underwent catheter ablation alone (7.9 ± 3.5 vs. 5.1 ± 2.1 s, *p <* 0.001) (Fig. 2). ROC curve analysis showed that the optimal cutoff point for predicting implantation of a permanent pacemaker following catheter ablation was 6.3 s (sensitivity 72.7%, specificity 79.1%, AUC = 0.75). The longest pause was associated with a need for implantation of a permanent pacemaker using both univariate analyses (HR 1.287, 95% CI 1.101-1.506, *p* = 0.002) and a multivariate model (HR 1.576, 95% CI 1.060-2.343, *p* = 0.025) adjusted by age, sex, time of AF symptom onset, HTN, DM, use of post-procedural AAD, LVEF, LA diameter, and trigger (Table 3). Antiplatelet therapy (aspirin, *n* = 1) was continued during pacemaker implantation. Anticoagulation therapy with warfarin (*n* = 9) was continued without heparin bridge during pacemaker implantation. One patient did not receive any antithrombotic therapy before the procedure. Among total 11 patients with pacemaker implantation after ablation, there was no pocket hematoma.

**Table 2:** Patients who underwent implantation of permanent pacemaker after RFCA

No.	Age (years)	Sex	Longest pause (seconds)	LA size (mm)	CHA_2_DS_2_ –VASc score	Trigger	AAD or NB after ablation	AF recur	Symptom after ablation	PM indication	Pause after ablation (seconds)	Time interval from RFCA to PM (days)	PM mode
1	71	M	10.1	39.5	1	None	None	recur	dizziness	SP	4.2	2422	DDD
2	60	F	5.2	49.5	1	None	None	SR	dizziness	SP	5.1	14	DDDR
3	67	M	12.8	37.8	1	LSPV	None	SR	syncope	SP	5.2	7	DDDR
4	49	F	13.6	47.9	1	SVC	None	SR	syncope	SP	8.5	70	DDDR
5	59	M	6.3	40.7	1	LSPV	None	SR	dizziness	SP	7.2	566	DDDR
6	52	F	6.9	36.0	2	SVC	None	recur	dizziness	SP	5.3	1504	DDDR
7	58	F	3.4	43.3	2	LSPV	None	SR	dizziness	SP	7.0	1269	DDDR
8	70	M	8.2	43.1	2	LSPV	None	SR	dizziness	SP	6.8	27	DDDR
9	61	F	7.2	60.8	2	None	None	SR	dizziness	SP	6.8	1126	DDDR
10	70	F	9.8	32.2	3	SVC	None	SR	dizziness	SP	5.2	51	DDDR
11	69	F	3.1	39.7	3	None	None	SR	dizziness	SP	4.7	250	DDDR

*No*. patients number, *M* male, *F* female, *RFCA* radiofrequency catheter ablation, *PM* pacemaker, *AAD* anti-arrhythmic drug, *NB* nodal blocker, *SR* sinus rhythm, *AF* atrial fibrillation, *SP* sinus pause

**Table 3:** Factors associated with pacemaker implantation after ablation

Factors	HR (univariate analysis)	*P* value	HR (multivariate analysis)	*P* value
Age, years	1.014 (0.954-1.078)	0.657		
Female sex	0.488 (0.142-1.676)	0.254		
Longest pause, seconds	1.287 (1.101-1.506)	0.002	1.576 (1.060-1.343)	0.025
Time of AF symptom onset, months	0.978 (0.933-1.025)	0.357		
AAD after ablation	1.459 (0.308-6.904)	0.634		
LVEF, %	1.024 (0.952-1.102)	0.525		
LA diameter, mm	1.086 (0.990-1.191)	0.080	1.230 (0.860-1.757)	0.257
Trigger (vs. no-trigger)	0.753 (0.217-2.614)	0.655		

Values are expressed as hazard ratios (HRs) with CI 95%. *AAD* anti-arrhythmic drug, *LVEF* left ventricular ejection fraction, *LA* left atrium

## Discussion

### Main findings

This study demonstrated that recurrence rate after catheter ablation were 19% in patients with AF predisposing to TBS during mean 29 months of follow up, 9.1% of patients were required implantation of a permanent pacemaker after catheter ablation, and they had a longer pause on termination of AF compared to those with catheter ablation alone. Multivariate analysis showed that a pause of 6.3 s or longer at baseline was associated with the need to implant a permanent pacemaker after catheter ablation.

### Mechanisms of TBS with AF

SND is frequently associated with AF [11], and is caused by inhomogeneous refractoriness [15]. A study in a chronic pacing-induced AF dog model demonstrated sinus node remodeling as a result of AF that was characterized by prolongation of corrected sinus node recovery time and P-wave duration and a decrease in maximal and intrinsic heart rate [2]. Sick sinus syndrome can be regarded as an atrial disease rather than as sinus node disease per se [16–18]. The mechanism of TBS, where the pause is manifested just after AF terminates, remains to be determined. Yeh et al. suggested that funny current (*I*_f_) down-regulation may contribute to the clinically significant association between SND and supraventricular tachyarrhythmias [19]. Recently, Duhme et al. demonstrated that altered C-linker interaction in hyperpolarized-activated ion channel HCN4 is associated with familial TBS and AF, indicating that funny channel dysfunction contributes to the development of atrial tachyarrhythmias [20]. Ectopic activities that elicit triggers for initiation of AF may also be induced in HCN4-K530 N by the switch from enforced inhibition of gating to stimulation of gating due to binding of cAMP under adrenergic stress. Furthermore, slow heart rates may increase susceptibility to ectopic beats. Therefore, molecular and structural remodeling of the sinus node increases arrhythmogenesis, promoting the vicious cycle of “AF begets AF” [21]. Thus, early ablation for TBS likely decreases the rate of implantation of permanent pacemakers before predisposition to SND by AF burden.

### Catheter ablation for TBS with AF

Catheter ablation has been used to treat patients with AF for several decades. Clinical outcome is better in paroxysmal AF than in persistent AF. The reasons for this include the lower severity of the remodeling process in paroxysmal AF than persistent AF, and the main cause of AF onset being ectopic beats that can be eliminated by catheter ablation. Catheter ablation may also improve sinus node function in patients with TBS and AF by inducing reverse remodeling. Hocini et al. demonstrated that successful ablation of AF was followed by marked recovery in sinus atrial node function when AF patients showed prolonged sinus pauses on AF termination [7]. These concepts suggest that TBS that manifests as trigger-AF may be cured by catheter ablation. Premature beat or activity originating from the PV is well-known and is the most common trigger in patients with AF [22, 23]. Miyazaki et al. demonstrated that SVC plays a role in AF not only as a trigger, but also as a perpetuator [24]. We identified triggers in 60.3% of patients. PV trigger activity was 86% and the most common non-PV trigger originated from the SVC (8 of 10). However, there was no significant difference in the rate of freedom from atrial tachyarrhythmia between PV trigger and non-PV trigger patients.

### Permanent pacemaker or catheter ablation?

Whether catheter ablation or implantation of a permanent pacemaker should be the first-line treatment remains debated. Prior to the AF-ablation era, pacing was the only option for treatment of TBS because tachycardia therapy using AAD aggravated bradycardia. Patients with drug-resistant tachycardia were considered to be candidates for catheter ablation even at that time [25]. However, the treatment strategy of pacemaker plus AAD has many weaknesses, including pacemaker- and AF-related problems. In patients who receive a pacemaker, various device-related complications may occur, such as infection, endocarditis, vascular injury, lead extraction, and pocket hematoma. In our study, there was no pocket hematoma in 11 patients who underwent pacemaker implantation. Recently, Malagù M et al. demonstrated that uninterrupted antiplatelet therapy or continued anticoagulation therapy without heparin bridge based on thromboembolic risk stratification was associated with a reduced incidence of clinically significant pocket hematoma [26]. The incidence of pocket hematoma was 1.6% in no-bridge protocol group and 6.5% in conventional management group. Pacing-induced heart failure may be a potential comorbidity. Need for generator change will increase as average life expectancy increases compared to device longevity. Because the risk of stroke increases in elderly patients with AF, use of certain diagnostic tools, such as magnetic resonance imaging, becomes problematic. Furthermore, AF still remains as a comorbidity in patients who undergo implantation of pacemaker. Moreover, AF itself may lead to medical problems (e.g. progression to persistent AF, tachycardia-mediated cardiomyopathy, proarrhythmic events due to uses of AAD, bleeding due to maintenance of antithrombotic therapy), and the management for AF is also needed indefinitely. In contrast to pacemaker implantation, catheter ablation of AF has several strengths in patients with TBS including eradication of AF and no need for a device. However, it was not clear whether catheter ablation or pacemaker implantation was better for treating paroxysmal AF-related TBS. Furthermore, recent studies demonstrated that maintenance of sinus rhythm following catheter ablation might reduce the risk of stroke compared with AAD therapy alone [27–29]. In our study, two patients were hospitalized due to stroke after catheter ablation. Further study is required to address whether ablation is more beneficial than implantation of permanent pacemaker for preventing stroke.

### Prediction for implantation of a pacemaker in TBS: TBS or intrinsic SND?

Miyanaga et al. reported that mean heart rate did not increase in TBS patients, probably due to pre-existing SND, although parasympathetic modulation was significantly attenuated after CPVI [30]. Inada et al. reported that a pacemaker was required in 8% of patients with paroxysmal AF and prolonged sinus pauses following catheter ablation, but gradual progression of SND occurred after long-term follow-up of over 3 years [8]. In our study, a pacemaker had to be implanted in 9.1% of patients who underwent catheter ablation due to bradycardia-related symptoms, such as syncope. Mean time interval from catheter ablation to implantation of a permanent pacemaker was 21 months, which suggests that intrinsic SND was progressive. Nevertheless, it is difficult to differentiate between patients who have AF with TBS or intrinsic SND because the characteristics of intrinsic SND are similar to those of TBS. We found that implantation of a permanent pacemaker after catheter ablation was required in patients with a long pause (≥ 6.3 s). This finding suggests that a pacemaker should primarily be considered in patients with a long pause on AF termination. Of course, SND might be caused or accelerated by the ageing process [31, 32]. However, this still remains unclear [33]. The ages of patients who received pacemaker implantation and those who did not after catheter ablation were similar. In addition, the rates of freedom from atrial tachyarrhythmia following catheter ablation were similar between patients who were 70 years or older versus those younger than 70 years. Recently, Nademanee et al. demonstrated that elderly patients with AF benefited from AF ablation, which was safe and effective at maintaining sinus rhythm and was associated with lower mortality and stroke risk [34]. Thus, the ageing process may not be the sole mechanism affecting the pathophysiology of TBS and AF.

### Study limitations

This was not a randomized trial that was designed to determine whether ablation was superior to pacemaker implantation. In this retrospective study, the decision of whether to perform catheter ablation or to implant a permanent pacemaker as the first-line treatment was at the physicians’ discretion based on clinical manifestations. Rates of recurrence of atrial tachyarrhythmias might also have been underestimated because we did not use a continuous rhythm monitoring device, such as an implantable loop recorder, for detection of AF [35].

## Conclusions

This long-term follow-up study of patients with TBS and AF showed that implantation of a permanent pacemaker after catheter ablation of AF may be required in patients who have a long pause on AF termination. Individualized treatment considering the length of pause when AF terminates is recommended in patients with TBS due to AF.

## Additional files

Additional file 1: **Figure S1.** There was no significant difference of atrial tachyarrhythmia recurrence between patients with triggers and those with no trigger (17.8% vs. 20.8%, log-rank test *p* = 0.559). (TIF 1067 kb)

Additional file 2: **Figure S2.** There was no significant difference of atrial tachyarrhythmia recurrence between patients with PV trigger and those with non-PV trigger (18.6% vs. 14.3%, log-rank test *p* =0.817). (TIF 1000 kb)

## Abbreviations

AAD: Antiarrhythmic drug
AF: Atrial fibrillation
AT: Atrial tachycardia
AUC: Area-under-the-curve
CI: Confidential interval
CPVI: Circumferential pulmonary vein isolation
CS: Coronary sinus
ECG: Electrocardiography
LA: Left atrium
LVEF: Left ventricular ejection fraction
NOAC: Non-vitamin K antagonist anticoagulant
RA: Right atrium
ROC: Receiver operating characteristic
SND: Sinus node dysfunction
SVC: Superior vena cava
TBS: Tachycardia-bradycardia syndrome
