# Supplementary figures and images for: Long-term clinical outcomes of catheter ablation in patients with atrial fibrillation predisposing to tachycardia-bradycardia syndrome: a long pause predicts implantation of a permanent pacemaker
Source: BMC Cardiovasc Disord. 2018 May 30;18:106. doi: 10.1186/s12872-018-0834-0 (PMC5975575; doi:10.1186/s12872-018-0834-0)

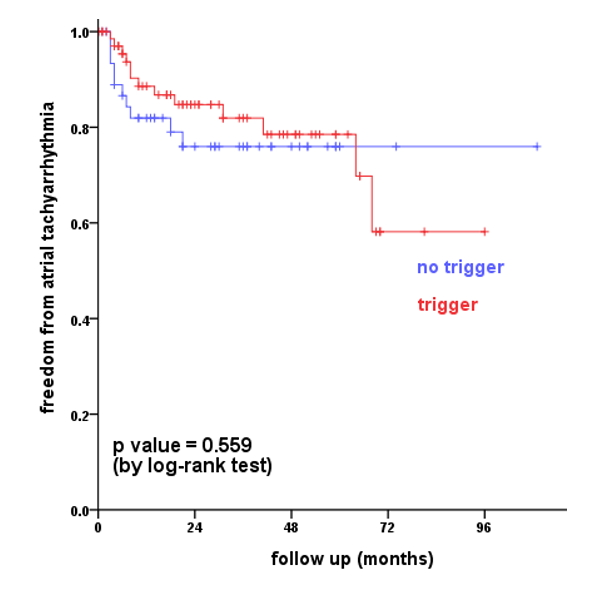

Supplement: Supplementary file 1 — Figure S1. There was no significant difference of atrial tachyarrhythmia recurrence between patients with triggers and those with no trigger (17.8% vs. 20.8%, log-rank test p = 0.559). (TIF 1067 kb) [file 12872_2018_834_MOESM1_ESM.tif]

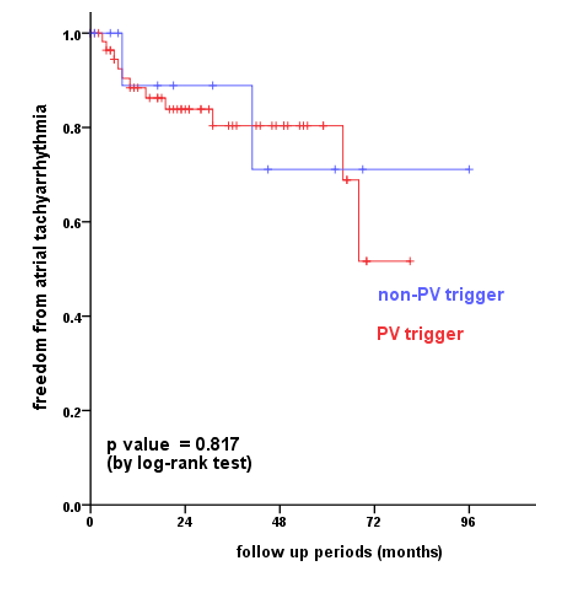

Supplement: Supplementary file 2 — Figure S2. There was no significant difference of atrial tachyarrhythmia recurrence between patients with PV trigger and those with non-PV trigger (18.6% vs. 14.3%, log-rank test p =0.817). (TIF 1000 kb) [file 12872_2018_834_MOESM2_ESM.tif]
